# Interaction of Obesity and Central Obesity on Elevated Urinary Albumin-to-Creatinine Ratio

**DOI:** 10.1371/journal.pone.0098926

**Published:** 2014-06-03

**Authors:** Nan Du, Hao Peng, Xiangqin Chao, Qiu Zhang, Honggang Tian, Hongmei Li

**Affiliations:** 1 Department of Epidemiology, School of Public Health, Medical College of Soochow University, Suzhou, China; 2 Center for Disease Prevention and Control of GuSu District, Suzhou, China; Temple University School of Medicine, United States of America

## Abstract

**Background:**

Microalbuminuria was much more common among obese individuals indicating a probable association with obesity. However, association of microalbuminuria with interaction between obesity and central obesity has not yet been studied.

**Design and Methods:**

A cross-sectional study was conducted in a 2889 general population aged ≥30 years. Obesity was defined as body mass index ≥28.0 kg/m^2^ and central obesity was defined as waist-to-hip ratio ≥0.85 for females and ≥0.90 for males. Both additive and multipliable interactions between obesity and central obesity on elevated urinary albumin-to-creatinine ratio (UACR) were evaluated.

**Results:**

After controlling for potential covariates, participants with both obesity and central obesity have significantly increased risk for elevated UACR (OR = 1.82 *P*<0.001) compared to those with neither. Additive interaction analysis indicated that about 43.9% of the risk of elevated UACR in participants with both obesity and central obesity was attributed to the interaction between obesity and central obesity (the attributable proportion because of the interaction: 0.439; 95% CI: 0.110–0.768). The multipliable interactive effect between obesity and central obesity on elevated UACR was not found significant (OR = 1.82, *P* = 0.078).

**Conclusions:**

Microalbuminuria was significantly associated with the interaction between obesity and central obesity. Our results indicated that individuals with both obesity and central obesity should be intensively managed to prevent renal diseases.

## Introduction

Microalbuminuria is recognized as an early sign of renal damage, and an independent predictor of end-stage renal disease [1], [2]. In addition to obesity and other conventional risk factors, microalbuminuria is recently treated as an independent risk factor for cardiovascular disease (CVD) [3]. Microalbuminuria has been reported to be prevalent in about 8.8–22% of general population [4], [5]. Previous studies found that microalbuminuria was much more common among obese individuals [4], which indicated a probable association between obesity and microalbuminuria. Several studies have revealed a significant association of microalbuminuria with either obesity or central obesity [6], [7]. However, no study focuses on the relationship between mircoalbuminuria and the interaction between obesity and central obesity. Thus, in addition to obesity and central obesity, the interactive effect between obesity and central obesity on mircoalbuminuria was investigated among a population aged over 30 years in Suzhou, China.

## Materials and Methods

### Study population

In this cross-sectional study in Suzhou city, Jiangsu province, China, we selected four urban communities and four rural villages using multi-phase cluster random sampling as investigation fields from January to May 2010. The inclusion criteria were as follows: (1) age ≥30 years; (2) ethnicity: Han; (3) no clinical evidence of end-organ damage at the screening visit [8]. The exclusion criteria were as follows: (1) clinical suspicion of urinary tract infection or gout; (2) receiving treatment of albuminuria within the last 2 weeks; (3) no history of chronic renal disease or tumors. There were 4076 adults met the inclusion criteria. However, only 3061 (participating rate: 75.1%) agreed to participate in the study. After excluding the participants with missing data, 2889 participants were eligible for the present analysis. This study was approved by the Soochow University Ethics Committee, and all participants provided written informed consent.

### Data collection

Data on demographic information, lifestyle risk factors, family history of hypertension, and personal medical history were obtained using a standard questionnaire in Chinese administered by trained staff. Body weight and height were measured by using a regularly calibrated stadiometer and balance-beam scale with participants wearing light clothing and no shoes. Body mass index (BMI) was calculated as weight in kilograms divided by square of the height in meters. Obesity was defined as BMI ≥28.0 kg/m^2^ and overweight was defined as 24.0 kg/m^2^ ≤ BMI ≤27.9 kg/m^2^ [9]. Waist circumference was measured at the level of 1 cm above the umbilicus. Hip circumference was measured around the widest portion of the buttocks, with the tape parallel to the floor. The waist-to-hip ratio (WHR) was calculated as the ratio of the circumference of the waist to that of the hips. Central obesity was defined as WHR ≥0.85 for females and ≥0.90 for males [10]. Three sitting consecutive blood pressure measurements (3 min between each) were taken by trained staff using a standard mercury sphygmomanometer according to a standard protocol, after the subjects had been resting for at least 5 min. The first and fifth Korotkoff sounds were recorded as systolic blood pressure (SBP) and diastolic blood pressure (DBP), respectively. The mean of three readings was used in analysis. Hypertension was defined as SBP ≥140 mmHg and/or DBP ≥90 mmHg and/or use of antihypertensive medication in the last 2 weeks [11]. Current cigarette smoking was defined as having smoked at least 1 cigarette per day for 1 year or more. Current drinking was defined as consuming alcoholic drinks once or more per week during the last 3 years.

Blood samples were obtained in the morning by venipuncture after a requested overnight fasting (at least 8 h). All plasma and serum samples were frozen at −80°C until laboratory testing. Total cholesterol (TC), triglycerides (TG), high-density lipoprotein-cholesterol (HDL-C), low-density lipoprotein-cholesterol (LDL-C), fasting plasma glucose (FPG) and serum uric acid (UA) were measured for all subjects. All the biochemical indexes were analyzed enzymatically on Hitachi 7020 automatic biochemical analyzer using commercial reagents. A 5-ml midstream urine specimen was obtained from a first-morning void urine for every subject and women who were actively menstruating were excluded from the urine test. Urine creatinine and albumin in fresh urine samples were determined within 24 h after collection in laboratory. Urine albumin was assessed using a nephelometric procedure with a specific antialbumin monoclonal antibody, and urine creatinine was assessed using the Jaffe method. Urinary albumin-to-creatinine ratio (UACR) was calculated as follows: UACR (mg/g)  =  urine albumin (mg/dl)/urine creatinine (g/dl). UACR was used to estimate urinary albumin excretion because UACR in a first-morning void urine could basically reflect average level of urinary albumin excretion in 24 h [12]. Furthermore, the National Kidney Foundation [13] recommended that UACR ≥17 mg/g for males and UACR ≥25 mg/g for females should be considered elevated.

### Statistical analysis

All subjects were divided into two groups: normal and elevated UACR. For the continuous variables of normal distribution (age, SBP, DBP, BMI and WHR), the means and standard deviations were calculated for the two groups. Comparisons in means between two groups were performed by using a Student-t test. For the continuous variables of skewed distribution (TG, TC, FPG, HDL-C, LDL-C and UA), medians (inter-quartile range) were calculated for the two groups. Comparisons in medians between two groups were performed by using Wilcoxon rank-sum test. The rates for categorical variables (gender, residence, hypertension, categories of BMI and WHR) were calculated for the two groups. Comparisons in the rates between two groups were performed by using the Chi-square test. Associations of elevated UACR with overweight or obesity and central obesity were evaluated using univariate and multivariate logistic regression models, respectively. In the multivariate model, variables that differed between participants with normal and elevated UACR such as age, gender, residence, smoking, alcohol consumption, hypertension, FPG, TG and uric acid were adjusted. Distribution of elevated UACR among participants with obesity and/or central obesity was assessed using the Chi-square test and trend of the prevalence of elevated UACR crossing groups was determined by the Chi-square trend test. Biological interactive effect between obesity and central obesity on elevated UACR was analyzed in both additive scale and multipliable scale using logistic regression analysis. The additive interaction analysis was performed by comparing participants with either obesity or central obesity and with both of them to participants with neither obesity nor central obesity. Biological interaction based on the additive interaction of obesity and central obesity was evaluated by 3 indexes: RERI, the relative excess risk because of the interaction; AP, the attributable proportion because of the interaction; and SI, the synergy index [14]. If there was no biological interaction, the confidence interval (CI) of RERI and AP included 0, and the CI of SI contained 1.0. The multipliable interaction was determined using the term of obesity×central obesity as an independent variable in logistic regression models. A two-tailed *P* value <0.05 was considered statistically significant. All statistical analyses were conducted using SAS statistical software (version 9.1, Cary, North Carolina, USA).

## Results

### Baseline characteristics

A total of 2889 subjects including 1116 males and 1773 females were studied in the current analysis, and their average age was 54.31 years. 613 (21.2%) participants were found to have elevated UACR. Table 1 shows the baseline characteristics of participants with normal and elevated UACR. Compared with participants with normal UACR, participants who had an elevated UACR were more likely to be older, female, nonsmokers and hypertensives, had higher levels of FPG, TG, TC, serum uric acid, SBP, and DBP. However, proportions of current drinkers and urban residents were not found different significantly between participants with normal and elevated UACR. In addition, BMI and WHR levels and proportions of overweight, obesity and central obesity were compared between the normal and the elevated UACR groups. Average levels of BMI (25.4 *vs.* 24.4, *P*<0.001) and WHR(0.89 *vs.* 0.87, *P*<0.001) were higher in participants with elevated UACR than in participants with normal UACR. Both obesity (21.4% *vs.* 13.3%) and central obesity (64.1% *vs.* 48.5%) were more common in participants with elevated UACR than in those with normal UACR (all *P* values <0.001).

**Table 1:** Baseline characteristics of participants with normal and elevated urinary albumin-to-creatinine ratio.

Characteristics	Normal UACR (*n* = 2276)	Elevated UACR (*n* = 613)	*P* value
Urban residents, *n* (%)	1350(59.3)	343(56.0)	0.134
Age, mean±SD	53.37±10.24	57.94±10.29	<0.001
Females, *n* (%)	1370 (60.2%)	403 (65.7%)	0.001
Current smoker, *n* (%)	516 (22.7%)	102 (16.6%)	0.001
Current drinker, *n* (%)	410 (18.0%)	97 (15.8%)	0.231
Fasting plasma glucose, mmol/L	5.20 (4.70–5.70)	5.40 (4.90–6.25)	<0.001
Total cholesterol, mmol/L	5.08 (4.49–5.71)	5.29 (4.71–5.98)	<0.001
Triglycerides, mmol/L	1.12 (0.79–1.62)	1.28 (0.85–1.79)	<0.001
Serum uric acid, mmol/L	268 (213–333)	276 (223–346)	0.026
LDL-cholesterol, mmol/L	2.95 (2.49–3.47)	3.13 (2.64–3.67)	<0.001
HDL-cholesterol, mmol/L	1.45 (1.22–1.72)	1.43 (1.21–1.68)	0.357
Systolic blood pressure, mean±SD	129.1±16.2	140.0±19.8	<0.001
Diastolic blood pressure, mean±SD	84.1±9.0	88.3±9.9	<0.001
Hypertension, *n* (%)	962(47.3)	406(66.2)	<0.001
BMI(kg/m^2^), mean±SD	24.61±3.55	25.70±4.47	<0.001
WHR, mean±SD	0.89±0.10	0.90±0.30	<0.001
BMI, *n*(%)			<0.001
<24	1024 (45.0)	204 (33.3)	
24.0–27.9	949 (41.7)	278 (45.3)	
≥28	303 (13.3)	131 (21.4)	<0.001
Central obesity, *n* (%)	1103 (48.5)	393 (64.1)	<0.001

Data are presented as median (interquartile range) unless otherwise noted. LDL: low density lipoprotein; HDL: high density lipoprotein; UACR: urinary albumin-to-creatinine ratio. Elevated UACR was defined as UACR ≥17 mg/g for males and ≥25 mg/g for females. BMI: body mass index. WHR: wait-to-hip ratio, central obesity was defined asWHR ≥0.85 for females and ≥0.90 for males.

### Obesity and elevated UACR

Associations of elevated UACR with obesity and central obesity were presented in Table 2. In the univariate logistic analysis, compared to participants with normal weight, participants with overweight (OR = 1.47, *P*<0.001) and obesity (OR = 2.17, *P*<0.001) had a significantly increased risk for elevated UACR, respectively. Similarly, compared to participants with normal WHR, participants with central obesity also had a significantly increased risk for elevated UACR (OR = 1.93, *P*<0.001). After adjusting for age, gender, smoking, alcohol drinking, residence, hypertension, blood glucose, triglyceride and uric acid, compared with the normal group, obesity (OR = 1.61, *P* = 0.001) and central obesity (OR = 1.31, *P* = 0.007) still increased the risk of elevated UACR, respectively, OR of elevated UACR in participants with overweight (OR = 1.16, *P* = 0.186) became no more statistically significant.

**Table 2:** Odd Ratios and 95% confidence intervals of elevated urinary albumin-to-creatinine ratio with obesity.

Variables	Univariate model		Multivariate model*	
	OR(95% CI)	P-value	OR(95%CI)	P-value
BMI(kg/m^2^)				
<24	1.00(reference)		1.00(reference)	
24.0–27.9	1.47(1.20–1.80)	<0.001	1.16(0.93–1.44)	0.186
≥28	2.17(1.68–2.80)	<0.001	1.61(1.22–2.13)	0.001
WHR				
female:<0.85,male:<0.90	1.00(reference)		1.00(reference)	
female:≥0.85,male:≥0.90	1.93(1.60–2.32)	<0.001	1.31 (1.08–1.60)	0.007

*Adjusted for age, gender, residence, smoking and alcohol drinking, hypertension, blood glucose, triglyceride and uric acid.

**Figure 1:**
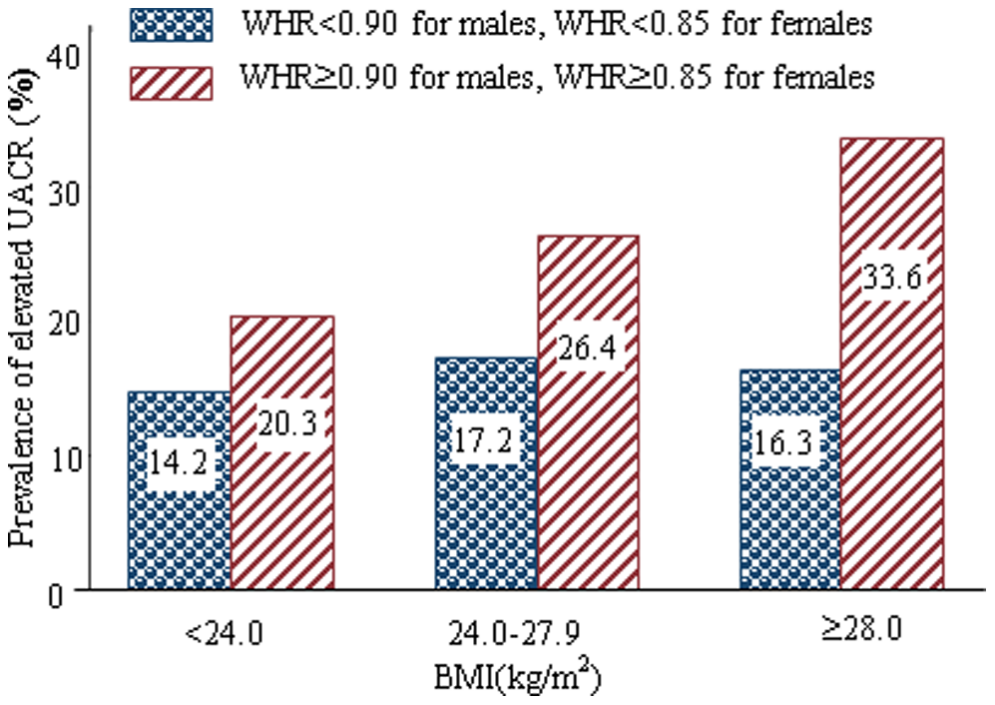
Distribution of elevated urinary albumin-to-creatinine ratio in obese and/or central obese participants.

All subjects were stratified into three categories by BMI, and then each category was divided into two groups: with and without central obesity, the prevalence of elevated UACR was calculated in each subgroup (Figure 1). In all BMI subgroups, prevalences of elevated UACR were significantly higher in participants with central obesity than those with normal WHR (all *P* values <0.001). Moreover, with the increase of BMI level, the prevalence of elevated UACR positively increased in participants with central obesity (*P*<0.001 for trend), which indicated a possible interaction between obesity and central obesity.

### Interaction between obesity and central obesity

Participants were further categorized into 4 study groups: participants with neither obesity nor central obesity, participants with isolated obesity,participants with isolated central obesity, and participants with both obesity and central obesity. As shown in Table 3, compared with participants with neither obesity nor central obesity, participants with just obesity or central obesity had no significant risks for elevated UACR after adjustment for multi-variables, and the adjusted ORs were 0.86 (*P* = 0.620) and 1.16 (*P* = 0.175) respectively, however participants with both obesity and central obesity had a significantly increased risk for elevated UACR, and the adjusted OR was 1.82 (*P*<0.001). Multipliable interaction in the term of obesity×central obesity on the elevated UACR were not found significantly in the univariate (OR = 1.52, *P* = 0.208) and multivariate logistic analysis (OR = 1.82, *P* = 0.078).

**Table 3:** Interactive effect analysis of obesity and central obesity on elevated urinary albumin-to-creatinine ratio.

Categories		Elevated UACR/*n*	Un-adjusted		Adjusted*	
obesity	Central obesity		OR (95%CI)	*P* value	OR (95%CI)	*P* value
(−)	(−)	206/1315	1.00(reference)		1.00(reference)	
(−)	(+)	276/1140	1.72(1.41–2.10)	<0.001	1.16(0.94–1.44)	0.175
(+)	(−)	14/86	1.05(1.07–1.99)	0.880	0.86(0.47–1.57)	0.620
(+)	(+)	117/348	2.73(1.83–2.92)	<0.001	1.82(1.36–2.43)	<0.001
Obesity×Central obesity		613/2889	1.52(0.79–2.89)	0.208	1.82(0.94–3.55)	0.078

* Adjusted for age, gender, smoking and alcohol drinking, hypertension, residence, blood glucose, triglyceride and uric acid.

Biological interaction based on the additive interaction of obesity and central obesity was evaluated (Table 4). Measures of RERI and AP were significant, and this indicated a significant additive interaction between obesity and central obesity. There was about 43.9% (95%CI: 11.0%–76.8%) of the risk of elevated UACR in participants with both obesity and central obesity attributed to the interaction between obesity and central obesity, even after adjusting for age, gender, smoking and alcohol drinking, hypertension, residence, blood glucose, triglyceride and uric acid.

**Table 4:** Indexes of additive biological interactive effect of obesity and central obesity on elevated urinary albumin-to-creatinine ratio.

Measure	Un-adjusted			Adjusted		
	Estimate	Lower	Upper	Estimate	Lower	Upper
RERI	0.960	0.073	1.847	0.798	0.119	1.476
AP	0.352	0.069	0.635	0.439	0.110	0.768
S	2.252	0.861	5.894	42.743	-	-

RERI, the relative excess risk because of the interaction; AP, the attributable proportion because of the interaction; S, the synergy index. If there was no biological interaction, the confidence interval of RERI and AP include 0, and the confidence interval of S contains 1.

## Discussion

The key findings of the present study were positive associations between obesity, and central obesity and elevated UACR, especially a significant interactive effect between obesity and central obesity on elevated UACR. We found a positive and significant increase in prevalence of elevated UACR with BMI levels in participants with and without central obesity, respectively. This indicated a possible interaction between obesity and central obesity. In order to examine the interaction between obesity and central obesity, both additive and multipliable interactions were analyzed. Though the multipliable interaction was not significant, the statistical and biological additive interaction were found significant even after multivariate adjustment. Moreover, measures of RERI and AP remained significant, which showed a significant additive interaction between obesity and central obesity. Biological additive interaction analysis indicated that about 43.9% of the risk of elevated UACR in participants with both obesity and central obesity was attributed to the interaction between obesity and central obesity. So, our findings suggested that individuals with both obesity and central obesity have an apparently increased risk for microalbuminuria.

Microalbuminuria, resulting from leakage of albumin across the glomerular podocyte filtration barrier into the urine, is known to be a marker of generalized endothelial dysfunction [15]. And many studies have also shown that microalbuminuria is a strong and independent predictor of progressive kidney disease, cardiovascular events and all-cause mortality in diabetic, non-diabetic, hypertensive persons, and even in the general population. As the chronic kidney disease has become a worldwide public health problem, elevated UACR is becoming an early significant marker of chronic kidney disease [16]–[19].

Obesity is a medical condition in which excess body fat has accumulated to the extent that it may have an adverse effect on health [20]–[22]. It is often defined by BMI, which is closely related to both percentage body fat and total body fat [23] and further evaluated in terms of fat distribution via the WHR and total cardiovascular risk factors [24]. At present, obesity is a global health problem with increasing prevalence in many parts of the world [25]. Some cross-sectional studies found that central obesity was associated with microalbuminuria [6]. Thoenes et al. [6] analyzed 20828 hypertensive out-patients and reported that in the multivariate analysis, an abnormal waist circumference (males: WC≥102 cm, females: WC≥88 cm), but not BMI appears to be independently associated with microalbuminuria (OR: 1.132, 95% CI: 1.039–1.233). Results from a study in 1321 healthy, normotensive Korean men [7] showed that abdominal obesity (WC≥90 cm) was an independent predictor of microalbuminuria with OR of 2.54(95%CI: 1.22–5.30). Another research in non-diabetic South Asian subjects also found the highest quartile of WHR had OR of 4.1(1.6–10.0) with microalbuminuria compared with the lowest quartile of WHR. Chen et al. [4] performed a population based study in China to investigate the prevalence of elevated UACR and its relationships with the components of the metabolic syndrome, they also found that central obesity had a significantly positive correlation with elevated UACR. However, our results demonstrated that not only obesity defined according to BMI and central obesity defined according to WHR was independently associated with elevated UACR, but also both obesity and central obesity increased the risk of elevated UACR.

The exact mechanisms involved in the link between obesity and the development of renal disease cannot be deduced from our current study. Most likely, this relates to a multifactorial complex pathogenesis, and the main reasons are as follows: visceral fat cells can secrete cytokines such as tumor necrosis factor alpha (TNF alpha), interleukin 6 (IL-6), and transforming growth factor (TGF) etc., these factors characterizing inflammation underlie the development of obesity-related disorders, and they can cause the endothelial dysfunction of kidney and glomerular lesions and increase the glomerular basement membrane permeability by autocrine or paracrine pathway, thus leading to urinary albumin excretion [26], [27].

The prevalence of elevated UACR in this population was 21.2%. Some studies have shown that early detection and treatment of microalbuminuria was effective to prevent progression and even to reverse renal disease [28], [29]. Therefore, it is necessary to screen and/or monitor microalbuminuria for the obese population, especially for those people whose BMI and WHR were abnormal at the same time, in order to prevent some microalbuminuria patients from losing the chance of early treatment.

There were some limitations in the present study. First, this was a cross-sectional study therefore a causal relationship between obesity and the risk of elevated UACR could not be established. Secondly, approximately 25% of the eligible population did not choose to participate in the study, which may have introduced some selection bias. However, there were several important strengths of our study deserved to mention too. This is the largest study to examine the association of microalbuminuria with interaction between obesity and central obesity in an Asian population. Further, important co-variables were measured and controlled in analysis of the study.

In summary, our study indicated that individuals with higher levels of BMI and WHR had higher risk of abnormal urine albumin, these data suggest the future benefit of weight loss in reduction of urinary protein excretion. Moreover, few observational studies also have shown that weight reduction through diet and/or exercise results in reduction of urinary protein excretion, both UACR and overt proteinuria [30], [31]. Future prospective cohort studies and clinical trials should be conducted to test the causal relationship between interaction of obesity and central obesity and the risk of microalbuminuria.
